# Could the use of a new novel bipolar radiofrequency device (Aerin) improve nasal valve collapse? A systematic review and meta-analysis

**DOI:** 10.1186/s40463-023-00644-7

**Published:** 2023-06-22

**Authors:** Manuele Casale, Antonio Moffa, Lucrezia Giorgi, Michelangelo Pierri, Rodolfo Lugo, Ofer Jacobowitz, Peter Baptista

**Affiliations:** 1 School of Medicine, Università Campus Bio-Medico di Roma, Via Álvaro del Portillo, 21, 00128 Rome, RM Italy; 2 Unit of Integrated Therapies in Otolaryngology, Fondazione Policlinico Universitario Campus Bio-Medico, Rome, Italy; 3 Department of Otolaryngology Head and Neck Surgery, Hospital San José, 64718 Monterrey, Mexico; 4 ENT and Allergy Associates, New York, NY USA; 5 Department of Otorhinolaryngology, Clínica Universidad de Navarra, Pamplona, Spain

**Keywords:** Radiofrequency treatment, Vivaer, Office-based procedure, Nasal valve collapse, NOSE score

## Abstract

**Background:**

Surgical treatment for nasal obstruction caused by nasal valve collapse requires a significant recovery period and risks of complications, while nasal dilators are uncomfortable. Recently, radiofrequency treatment of lateral walls has been used under local anesthesia as an office base surgery. This work aims to assess the efficacy of a new radiofrequency device, the Vivaer™ System (Aerin Medical, Sunnyvale, CA), to treat nasal obstruction through a systematic review and meta-analysis.

**Methods:**

Two researchers independently reviewed the literature up to December 2021. Studies on patients seeking treatment for nasal obstruction due to nasal valve collapse were included in the analysis.

**Results:**

Four studies (218 patients) met the inclusion criteria and treated the nasal valve regions bilaterally with the Aerin Medical Vivaer™ System. After the treatment, the NOSE score was reduced at three months postoperatively. Minor adverse events were reported in the included studies, and two showed no complications. None of the studies reported changes in the external appearance of the nose.

**Conclusion:**

The radiofrequency treatment using the Vivaer device can be useful for treating nasal valve collapse, improving significantly subjective breathing symptom scores. Further studies on a large scale are needed to confirm these results.

## Introduction

Nasal obstruction is a highly prevalent, multifactorial disorder, which may be caused by several factors such as a nasal septal deviation, turbinate hypertrophy, rhinosinusitis with and without nasal polyps, allergic or nonallergic rhinitis, and last but not least, nasal valve collapse. Recently, there has been a great interest in managing nasal valve collapse. The nasal valve region is defined by the caudal cartilaginous nasal septum, the anterior head of the inferior turbinate, and the caudal end of the upper lateral cartilage. It represents the narrowest part of the nasal airway and is crucial in developing nasal obstruction [1–4]. Poiseuille’s law describes that minimal changes in the diameter of a tube (nasal cavity) result in exponential airflow changes, leading to nasal obstruction. Usually, patients with nasal valve collapse complain of nasal obstruction, headache, sleep disturbance, daytime sleepiness, and snoring. These symptoms can also influence many daily and social activities, worsening the quality of life. Several treatment options for nasal valve collapse include external/internal nasal dilators, surgical rhinoplasty, and/or nasal valve repair [5].

For patients who are poor surgical candidates or hesitant about surgery, there are nonsurgical management options for nasal valve collapse. Various nasal strips act on the lateral wall to strengthen and relieve the propensity for nasal valve collapse by expanding the internal nasal valve. As an alternative, internal dilators can be commercially purchased and, when placed within the nasal passage, act to stent the airway [6].

There are many brands and devices, but all act on either splinting the airway externally by supporting the lateral wall and internal nasal valve or internally by stenting the internal and external valves.

Multiple surgical options are available to open the internal nasal valve to improve breathing and alter the aesthetic appearance of the nose at the middle nasal vault. This can be accomplished by traditional spreader grafts, spreader flaps, butterfly grafts, and with additional support by batten grafts [7]. The most commonly used method to improve obstruction at the internal nasal valve is spreader graft placement. However, the inferior turbinate reduction is becoming increasingly prominent, which is discussed later in this article [8].

Also, surgical treatment usually requires a significant recovery period and risks of infection, bleeding, scarring, and graft complications, while nasal dilators are uncomfortable having to be worn daily.

Radiofrequency (RF) energy has been used for decades for several applications in otolaryngology, neurosurgery, cardiology, urology, and general surgery. In particular, RF has been used for turbinate reduction producing a thermal injury within the submucosal tissue of the turbinate, reducing tissue’s volume with minimal impact on surrounding tissues. Many studies showed that this technique could be safe and effective in improving nasal obstruction and inpreserving nasal function [9, 10]. Recently, RF has been applied for the treatment of lateral wall for patients with nasal valve collapse under local anesthesia without impacting nasal aesthetics as an office base surgery. In particular, a new RF device named the Vivaer™ System (Aerin Medical, Sunnyvale, CA) may create a thermal lesion in the submucosa of the nasal valve, resulting in tissue volume reduction and a slight expansion in the nasal valve [11, 12].

The aim of this work is to assess the efficacy of using a temperature-controlled RF treatment (Aerin Medical Vivaer™ System, Sunnyvale, CA) to treat nasal obstruction through a systematic review and meta-analysis.

## Materials and methods

### Search strategy and selection of studies

Studies published until December 2021 were identified from PubMed, SCOPUS, EMBASE, Web of Science, and Cochrane Database using the search terms: “radiofrequency device”, “Vivaer”, “minimally invasive surgery”, “Office-based procedure”, and “Nasal valve collapse”.

Two reviewers, working independently, screened all abstracts and titles for candidate studies and discarded studies unrelated to the RF device used for nasal valve collapse. Full texts of potentially relevant studies were obtained if a decision for selection could not be made from the abstract. Prospective or retrospective studies that met the following inclusion criterion were eligible for review: studies on patients seeking treatment for nasal obstruction due to nasal valve collapse with high Nasal Obstruction Symptom Evaluation (NOSE) scores (more than 55). However, studies were not eligible if patients underwent additional procedures such as septoplasty, turbinoplasty, rhinoplasty, and orthognathic surgery.

**Fig. 1:**
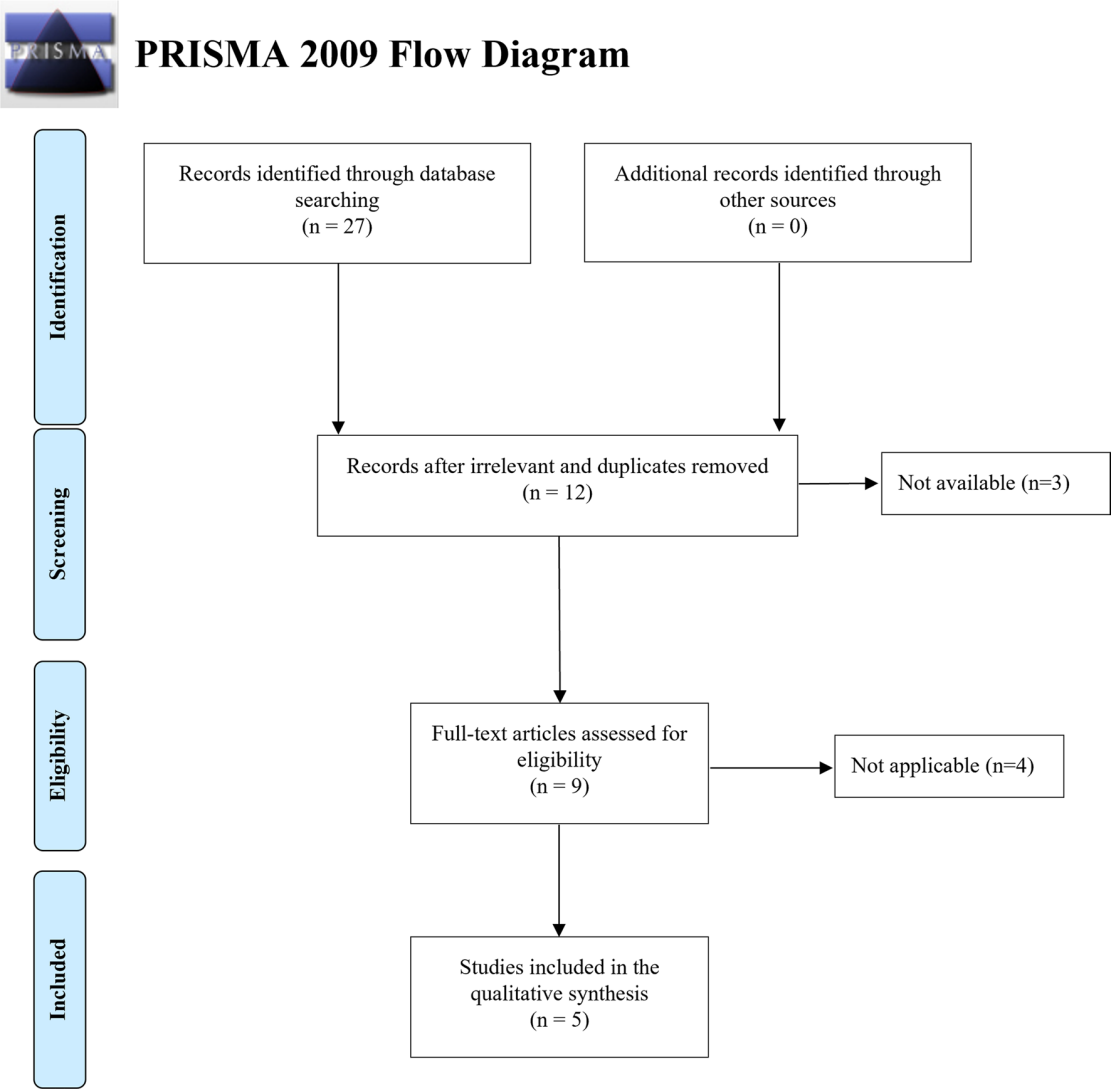
Flowchart outlining the paper selection process of the systematic review (based on PRISMA guidelines)

In addition, studies were excluded from analysis if they did not clearly report outcomes of interest with quantifiable data or if data could not be extracted or outcomes calculated from published results. The search strategy used to identify the studies selected for the meta-analysis is summarized in Fig. 1.

### Data extraction and risk of bias assessment

All articles were initially screened by title and abstract. The full-text version of each publication was assessed, and those whose content was judged not strictly related to this review's subject were excluded. Data extraction of the studies included the population demographics and baseline characteristics, details on intervention and control conditions, study designs, and outcomes. The outcome analyzed was the NOSE questionnaire, representing the disease-specific quality of life reported by patients, comparing pre-treatment and post-treatment values during the follow-up period. Severity was classified as follows: mild (5–25 points), moderate (30–50 points), severe (55–75 points), or extreme (80–100 points). Comparisons were analyzed between pre-treatment and post-treatment values, and/or between post-treatment and control (sham) outcomes during the follow-up period.

Additionally, adverse effects of the RF surgical procedure during the postoperative period (within 12 months) were analysed. For the incidences of side effects, considering that patients would undergo several implantations to alleviate the symptoms, the number of side effect events divided by the number of RF procedures is considered a more practical measurement of the side effect rate.

Lastly, for the non-randomized trial, the risk of bias was assessed using the ROBINS-I tool (Risk Of Bias In Non-randomized Studies—of Interventions), evaluating the risk of bias in the results of non-randomized studies of interventions (NRSI), including cohort studies, case–control studies, controlled before-and-after studies, interrupted-time-series studies and controlled trials in which intervention groups are allocated using a method that falls short of full randomization (sometimes called “quasi-randomized” studies) [13]. For the randomized trial the Cochrane Risk of Bias (RoB2) tool was used.

### Statistical analysis

A meta-analysis of the selected studies using continuous measures (comparison of means and standard deviations) was performed with the R statistical software version 4.1.2. The mean difference (MD), defined as the difference between the means of the NOSE score pre- and post-treatment, was chosen to represent the effect size when all studies used the same outcomes and units of measure. MD was calculated through the *meta* package. In addition, the test for heterogeneity was calculated using the I^2^ statistic, describing the percentage of variation across studies originating more from heterogeneity than from chance. The I^2^ rank was classified as follows: 0–24%, no heterogeneity; 25–49%, low heterogeneity; 50–74%, moderate heterogeneity; and 75% or more, high heterogeneity.

## Results

Search criteria returned 27 articles, then 18 were removed as irrelevant or duplicates. These were screened, and a further 4 were excluded, resulting in 5 articles fulfilling the inclusion criteria, all published in the last three years. All the original articles included were prospective clinical studies. The population in the included studies consisted of 297 patients aged between 19 and 83 years old. The baseline characteristics of the studies included are shown in Table 1. The authors followed the same protocol: the nasal valve regions were treated bilaterally with an office-based procedure under local anesthesia using Aerin Medical Vivaer™ System. Bilateral temperature-controlled RF treatment was applied in a single office visit using the Vivaer device at 60 °C and 4 W. The stylus treatment tip was positioned onto the mucosa overlying the lower edge of the upper lateral cartilage, and 3–4 non-overlapping areas on the lateral wall of the nasal valve were treated for 18 s on each side. Tissue temperature was maintained by feedback from the stylus at the treatment temperature of 60 °C. One study [14] compared the results obtained before and after treatment in the active group (RF treatment) with the ones obtained in the control group (sham procedure). During the sham procedure, the stylus was applied after local anesthesia injection in the same manner as for the active group but without RF energy delivery while audible tones mimicking activation of the Aerin Console were played. In the other four studies [11, 12, 15, 16] the authors did not perform a sham procedure.

**Table 1:** Summary of studies included in the meta-analysis

Author (Year)	Sample size	Diagnosis	Comparison	Outcome measured	Nose
Brehmer et al. [11]	31	Positive reaction to a minimum of two of four diagnostic tests: (1) Application of dilatative nasal strips like breath right (2) Modified Cottle test using a Q-tip (3) Use of nasal stents (4) Cottle test	Before treatment vs after treatment	NOSE SNOT 20 Mean snoring intensity (dB) Snore Outcomes survey EuroQoL group questionnaire for generic quality of life AHI Percent of sleeping time spent with a snoring intensity > 45 dB	Baseline: 65 ± 14.81 3-month: 30 ± 18.52
Jacobowitz et al. [12]	50	Positive symptomatic improvement with use of external or internal nasal dilators, Q-Tip or curette test (manual intranasal lateralization), or the Cottle Maneuver (manual lateral retraction of the cheek)	Before treatment vs after treatment	NOSE Satisfaction survey	Baseline: 79.9 ± 10.8 3-month: 27.3 ± 18 6-month: 24.7 20.4
Wu et al. [15]	20	Positive response to one of the following temporary measures: Breathe; Right Strips; Nasal stents; or modified Cottle maneuver	Before treatment vs after treatment	SNOT 22 NOSE VAS Volume of frontal 1/3 of nasal cavity (cm^3^) Peak heat flux posterior to vestibule	Baseline: 78.89 ± 11.57 3-month: 31.39 ± 18.30
Silvers et al. [14]	117 (active group: 77; control group: 40)	Positive response to a temporary nasal dilation measure (modified Cottle maneuver)	Before treatment vs after treatment Active group vs control group	NOSE Ease-of-breathing VAS score	Active group: baseline: 76.7 ± 12.6 3-months: 34.4 ± 24.85 Control group: baseline: 78.7 ± 14.3 3-months: 62.0 ± 29.04
Yao et al. [16]	119	Positive response to modified Cottle maneuver or other temporary nasal valve dilation or stabilizing measures	Before treatment vs after treatment	NOSE Satisfaction survey	Baseline: 80.3 ± 12.6 3-month: 32.9 ± 24.2

### Risk of bias

**Fig. 2:**
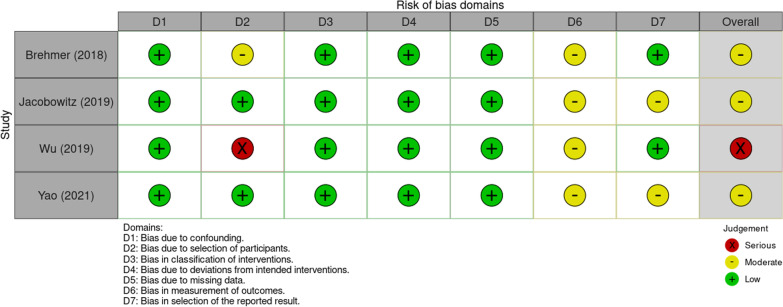
Risk of bias assessment

The risk of bias assessment for the four non-randomized trial is shown in Fig. 2. All the studies showed a low risk of bias due to confounding, classification of interventions, deviation from intended interventions, and missing data. A moderate risk should be considered for bias due to measurement of outcomes since outcome assessors are aware of intervention status. Also, moderate risk of bias due to selection of participants was found in Brehmer et al. [11] since the study had a duration of 9 months and it was not specified whether all subjects were enrolled during the same period. In the same domain, a severe risk of bias was found in Wu et al. [15] because the authors did not specify the enrolment period. A moderate risk of bias due to selection of reported results was found in two studies [12, 16] in which the authors analysed only the NOSE score and the satisfaction of the patients. Overall, three studies [11, 12, 16] showed a moderate risk of bias and one [15] serious.

The randomized clinical trial [14] showed a low risk of bias in all the domain (Randomization process, Missing outcome data, Measurement of the outcome, and Deviations from intended interventions) except for the “Selection of the reported result” domain, since the authors reported only NOSE outcomes, and so some concerns should be considered. Lastly, it should be taken into consideration that Wu et al. [15] and Silvers et al. [14] were funded by Aerin Medical Inc. for their trials. However, Wu et al. [15] declared that the funders had no role in data collection and analysis, decision to publish the manuscript.

### Changes in NOSE scores after treatment

**Fig. 3:**
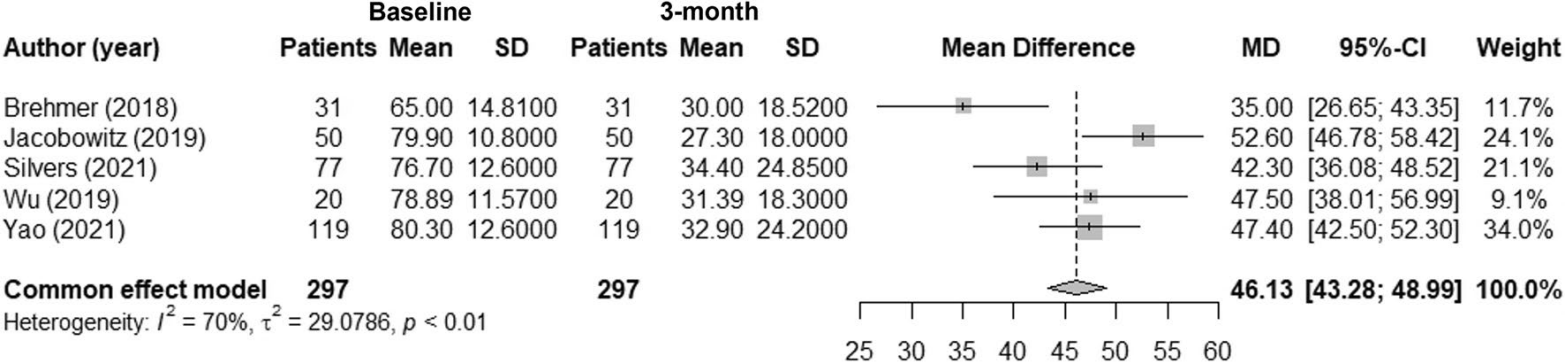
Mean pre-treatment and post-treatment differences in NOSE score at three months

Three months after treatment, NOSE scores reduced significantly (pre-treatment: 76.16 ± 6.39; post-treatment: 31.20 ± 2.73; MD: 46.13; 95% confidence interval [CI] 43.27–48.99) [11, 12, 14–16] with moderate heterogeneity (*I*^2^ = 70.1%) (Fig. 3). In the only randomized controlled study, the active group showed significantly better results than control group 3 months after treatment (active group from 76.7 ± 12.6 to 34.4 ± 24.8 vs control group from 78.8 ± 14.3 to 62.0 ± 29.04) [14].

### Adverse events after treatment

Reported adverse effects caused by RF treatment (the number of adverse effects/ the number of applications of RF device) in enrolled studies were nasal congestion, swelling, and mild pain limited to the first month (no previous adverse effect numbers specified) [12, 14]. Other adverse effects were pharyngitis (4%, 2/50) [12], vasovagal reaction (1.02%, 2/196) [14, 16], and intermittent nasal bleeding with mucus (1.30%, 1/77) [14]. Persistent symptoms at one month after the procedure were soreness of the nose (6%, 3/50) and crusting (34%, 17/50) [12]. In one study [16] the authors noted eight reports of nasal/sinus tenderness, crusting, and/or pressure/congestion (seven moderate and one severe, 6.72%, 8/119), all resolved during the study period. Also, in the same study one patient experienced a moderate sinus infection after the procedure, which resolved with antibiotic treatment [16]. No major complications occurred in two studies [11, 15]. None of the studies showed changes in the external appearance of the nose.

## Discussion

In this study, we investigated the RF effectiveness using the Vivaer device by systematically reviewing the current literature and a meta-analysis of relevant articles.

The Vivaer intranasal remodelling treatment is a minimally invasive procedure that uses a stylus to deliver targeted low-energy RF heating to the nasal sidewall to reshape the tissues. The device consists of a handle, shaft, and treatment tip and delivers temperature-controlled, low-energy RF to the target tissue. Adverse effects following nasal RF surgical procedures were resolved without sequelae and occurred infrequently. The Vivaer intranasal remodelling treatment significantly improved NOSE scores at 3 months postoperatively compared to pre-treatment [11, 12, 14, 15]. Indeed, we noted a significant improvement in NOSE score (pre-treatment values: 76.16 ± 6.39; post-treatment: 31.20 ± 2.73; MD: 46.13) almost ten time higher than the minimum important difference previously described by Ziai et al. [17] and comparable with the values found in literature [18]. In addition, RF surgical procedure significantly improved NOSE score compared with sham surgeries [14].

To the best of our knowledge, this is the first meta-analysis to assess the effectiveness of a specific temperature-controlled RF device to treat nasal valve collapse in the literature.

The internal nasal valve is the narrowest part of the nasal airway and provides approximately two-thirds of its resistance to airflow. Dynamic collapse or stenosis of the valve is a recognized but often overlooked cause of symptomatic nasal obstruction.

There are several diagnostic procedures to assess nasal valve collapse: Cottle's and modified Cottle’s tests. The first one is a test in which the cheek on the side to be evaluated is gently pulled laterally with one to two fingers to open the internal nasal valve. This test was used to determine if the most significant site of nasal obstruction was at the internal nasal valve or farther inside the nasal cavity. In Modified Cottle's Test, a Jobson’s Ring probe gently lateralizes the upper lateral wall cartilage on each side of the nose while the patient breathes. The test is positive if the patient appreciates significant improvement while breathing during inspiration [19].

Treatment using mechanical dilating devices such as external strips or intranasal inserts requires repeated application and is not always tolerated. Surgical treatments often require general anesthesia with extensive dissection and graft harvest and the risk of relapse, scarring, and postoperative external deformity.

The most critical structure for maintaining the strength of the lateral wall of the nose is the caudal border of the lower lateral cartilage. This area corresponds to the internal nasal valve. Cartilage, mainly filled with fibrous fatty tissue, often exists to strengthen the lateral wall of the nose. In nasal airway mucosa, an underlying network of collagen and elastin fibres provides scaffolding for the mucosa and determines its firmness and elasticity. RF-induced heating has been shown to induce tissue tightening through effects on this fibre network. Heating by RF energy causes two main effects on nasal airway tissue: contraction and tightening, through the immediate impact on existing collagen proteins and the induction of new collagen production. The device was designed to cause tissue-tightening effects within the submucosal layer of the nasal valve. The tightened submucosal layer likely accounts for the immediate and long-term contour changes in the treatment area, which results in greater airflow [20]. Another potential mechanism has been described, as cartilage shape can be altered using concurrent pressure and heat [21, 22].

An essential advantage of this minimally invasive technique is the possibility of performing the procedure both in the operating room with septoplasty and/or turbinate surgery, and in the clinic setting, as a stand-alone procedure or in conjunction with turbinate reduction [23]. Since this minimally invasive surgical procedure could reinforce the lateral wall over an extensive period, is easy to conduct, and is associated with a low incidence of complications, it is likely to become a staple procedure for addressing nasal obstruction. Recently an absorbable nasal valve implant has been proposed deployed via a hollow cannula to treat nasal valve collapse with a reduction of NOSE scale mean from 77 to 35 with a standard deviation of 29 at 12 months [24]. While this is a simple, rapid procedure, potential issues include improper placement, cosmetic changes, migration, foreign body sensation or reaction, and need for removal. It also requires alar rim anesthesia for placement in the office.

Only minor adverse events were shown in the included studies. In addition, none of the studies showed changes in the external appearance of the nose after the treatment.

Given the moderate heterogeneity of the results and the limited number of studies investigating small populations with short follow-up periods, the outcomes of this review must be considered with caution. This procedure is one of several non-surgical procedures suggested for the collapse of the nasal valve. It is a minimally invasive procedure associated with less pain, a shorter hospital stays, and fewer complications. More extensive comparative studies and well-designed randomized clinical trials with validated patient-reported outcome measures are required to provide more definitive recommendations.

## Conclusions

The RF treatment using the Vivaer device seems to be useful for treating nasal valve collapse, significantly improving subjective breathing symptom scores (NOSE) compared to preoperative status and control procedure. It is an easy-to-use, effective, and safe device that can be combined with other surgical procedures such as septoplasty and/or turbinate surgery. However, there are few and small studies on this topic. Further studies on a large scale assessing the role of this new RF device in reducing nasal valve collapse are needed to confirm these promising results.

## Data Availability

The datasets generated during and/or analysed during the current study are available from the corresponding author on reasonable request.

## Abbreviations

RF: Radiofrequency
NOSE: Nasal Obstruction Symptom Evaluation
MD: Mean difference
